# Supplementary material for: Determining the effects of nanoparticulate air pollution on proteostasis in Caenorhabditis elegans
Source: PLoS One. 2020 Dec 3;15(12):e0243419. doi: 10.1371/journal.pone.0243419 (PMC7714337; doi:10.1371/journal.pone.0243419)
Supplement: Attachment — Submitted filename: Review_PONE-D-20-23642.docx [file pone.0243419.s001.docx]

The Registered Report Study by Green and Kikis titled “Determining the effects of nanoparticulate air pollution on proteostasis in *Caenorhabditis elegans*” explores the impact of air pollution on organismal proteostasis. Nano-sized particulate matter (nPM) is known to contribute to the pathogenesis of neurodegenerative diseases across mouse models and in humans. Moreover, previous research using mice and *C. elegans* suggests that nPM affects host proteostasis. The authors pose to investigate the effect of nPM on the host by posing an interesting hypothesis “that exposure to nPM will challenge the buffering capacity of the proteostasis network, thereby reducing the efficiency of disease-associated protein folding.” *C. elegans* is an ideal model organism to test this hypothesis as it offers a comprehensive collection of neurodegenerative disease models, proteostasis reporters, and genetic tools. Moreover, the model lacks inflammatory response and can therefore be used to study a direct effect of nPM on proteostasis. Overall, the authors propose experiments that will explore a novel area of research that has been recently gaining interest within the scientific community. If successful, the results of this study can be extrapolated to and followed up in higher organisms. This study will provide exciting results and is recommended for publication in PLoS One. There are minor comments that need to be addressed:

- Why is nano-sized particulate air pollution abbreviated as nPM? What does M stand for? Missing “matter”?
- The threshold of polyQ aggregation is between 35-40. The authors may benefit by including polyQ37 strain instead of, or in addition to, polyQ40.
- It is assumed that the animals will ingest nPM, and the immediate effect will be on the proteostasis in the intestine. The study may benefit from including intestinal polyQ44. If the particulates are restricted to the intestine, muscle-specific polyQs may not be affected; however, that would not mean there is no effect.
- Are the nPM samples sterile? If not, will they be sterilized by UV, heat, or any other method? If not sterile, the bacteria in the sample may affect proteostasis.
- Line 108: change the citation style to numerical so it matches the rest of the paper.
- Line 108: with deionized water?
- If the stock nPM concentration is 150ug/mL and the animals will be exposed to 75ug/mL that means the stock will have to be diluted 1:1 to obtain the final concentration. The protocol states that 200uL of M9 supplemented with 10ug/mL cholesterol will be present in each well. It may be more clear to write that 100uL of 2X M9 at 20ug/mL cholesterol will be diluted with an equal volume of nPM.
- For aggregate counting, will only one biological replicate be performed?
- For qRT-PCR, it would be good if the authors could include mitochondrial hsp70 (hsp-6) as well. That way, all major stress pathways are assessed.
- Line 174: please add particulates “…exposure to air pollution particulates.”
- The authors have to be cautious about interpreting aggregate counts when normalized to body size. Although the animals may be smaller in size, they are not developmentally delayed (post L4) and their biological age does not change. The absolute numbers of aggregates may be a better approximation of the actual effect of nPM.
- Line 198: Please write out standard error of the mean and then abbreviate.
- The timeline seems appropriate
