# Supplementary material for: Determining the effects of nanoparticulate air pollution on proteostasis in Caenorhabditis elegans
Source: PLoS One. 2020 Dec 3;15(12):e0243419. doi: 10.1371/journal.pone.0243419 (PMC7714337; doi:10.1371/journal.pone.0243419)
Supplement: Attachment — Submitted filename: Response to Reviewers_Kikis.docx [file pone.0243419.s002.docx]

**Response to reviewers**

Dear Editor,

I would like to thank you for taking the time to handle the peer review process for our manuscript titled “Determining the effects of nanoparticulate air pollution on proteostasis in *Caenorhabditis elegans*” and for the thoughtful reviews and comments that we received. As requested, I have carefully considered all of the comments and concerns and have addressed them in the text to the extent that I deemed appropriate and feasible. This is my first foray into Registered Report Protocols, and feel that the process has done exactly what is intended. Specifically, it has been a pleasure to be able to share our ideas with the reviewers and receive suggestions aimed at strengthening our research plan. The resultant plan is one that, after receiving feedback, I am even more excited to embark on.

Changes and additions to the text are described in detail below:

1. **Reviewer #1 pointed out that we did not define the letter “M” in “nPM.”** This has been corrected. Specifically, we have included the following sentence in the introduction “…nano-sized particulate matter (nPM) obtained from traffic-derived air pollution…”
2. **Reviewer #1 suggested that we “may benefit by including polyQ37 instead of, or in addition to, polyQ40.”** This was an interesting suggestion and one that we seriously considered. In the end, we would like to stick to polyQ35 and polyQ40. This is because the polyQ40 strain has already been shown to be highly sensitive to changes in the protein folding environment. Namely, Gidalevitz et. al. demonstrated this is in *Science* 311 (5766), 1471-1474. More recently, this same protein was shown to be sensitive to changes in the genetic background *BMC biology* 18 (1), 1-20. Taken together, we think that polyQ40 is a very important strain to use in our proposed project. Fewer studies have utilized polyQ37. While we could theoretically use all three strains, we feel that that would be excessive and would needlessly use up too much of our stock of nPM, especially as we are also including in our study a strain expressing polyQ44 in the intestine and a strain expressing Abeta in body wall muscle cells.
3. **Reviewer #1 asked whether the nPM samples are sterile.** Based on this comment, we have included the following sentence in the methods section, “As bacterial contamination could affect proteostasis, nPM samples will be sterilized by UV-C irradiation in a biosafety cabinet for at least 15min. To ensure that samples are free of bacterial contamination, 20uL of nPM will be transferred to an NGM plate, incubated for 3 days and examined for the appearance of colonies.”
4. **Reviewer #1 suggested that we clarify the way our nPM will be diluted to obtain the appropriate final concentrations of nPM and Reviewer #2 also asked about the solvent for nPM and negative controls (I am paraphrasing these comments/suggestions):** To address these concerns, we have added the following sentence to the methods section, “Exposures will be performed at 20°C in 96 well plates. 100μL of 2X M9 liquid medium will be diluted with an equal volume of nPM (or water for unexposed controls) supplemented with 10μg/mL cholesterol and OP50 bacteria for up to 3 days.” It should be noted that nPM is already in water, so there are no solvent considerations beyond those addressed in the sentence above.
5. **Reviewer #1 asked whether only one biological replicate will be performed for aggregate counting.** All experiments in this study will be performed in at least biological triplicate. We have now clarified this throughout the text (in the section on aggregate counting and other sections as well).
6. **Reviewer #1 cautioned us not to normalize aggregate counts to body size.** Thank you for this suggestion. The text has been revised to remove any normalization to body size.
7. **Reviewer #2 expressed concern about the organization of the study. Specifically, this reviewer suggested that “establishing nPM-induced phenotypic changes should precede the mechanistic investigation of chaperone gene expression.”** This point is well-taken and we have rearranged the aims accordingly.
8. **Reviewer #2 stated that “aggregates simply cannot be counted by eye on a stereo-microscope.”** To address this concern, we have now indicated that “Aggregates will be counted in individual live animals using a fluorescent stereomicroscope (Leica M165 FC) fitted with a digital camera according to established methods (20).” The polyQ aggregates are very large, bright, and defined. Stereomicroscopes, albeit fitted with nice digital cameras, have been used successfully in many studies to quantify aggregate number. These studies include the original paper in which the muscle polyQ model was studied (Morley et. al., (2002) PNAS) and a more recent report of the effects of genetic background on polyQ aggregation (Alexander-Floyd et. al. (2020) BMC Biology). The methods we are proposing here mirror those of the more recent study.
9. **Reviewer #2 suggested that we “consider video capture of thrashing worms followed by automated analysis of multiple motor parameters.”** We are aware that recent technological advances have made such automated analysis increasingly common. If cost were not prohibitive, we would (gladly and enthusiastically) purchase a MicroTracker or similar device. However, at $16,400, this far exceeds our budget. Fortunately, our lab and others have utilized manual counting of thrashing effectively to observe changes in motility for these and similar animals. Thus, we are cautiously optimistic that, even with our proposed methods, we will be able to observe decreases in muscle function.
10. **Reviewer #2 suggested that “SDS-PAGE should be performed… in order to resolve SDS- and heat-stable aggregate species.”** We have now included this in the section on the characterization of large, visible, protein aggregates. Specifically, we state “As an additional assay for changes in the relative concentration of large visible aggregates in response to nPM, SDS-PAGE gels will be performed as described above. This is expected to reveal the formation of SDS-insoluble protein species.”
11. **Reviewer #2 suggested that we “be careful not to give the impression that the mechanisms of HD and AD are entirely known” and also “not overstate the evidence for the link between air pollution and AD onset being ‘well-established.’”** These are good points and the introduction has been revised accordingly.
12. **Reviewer #2 suggests that “it is not clear from the introduction precisely what is still not know regarding the effects of nPM on proteostasis.”** Thank you for mentioning that, because this is a very important point. In fact, very little is known about the effects of nPM on proteostasis and that is why we think that this study is so important. Specifically, there have only been two studies that touch on this at all and neither examined the effects of nPM on protein folding. To clarify this point, we have revised the introduction to now say, “Consistent with this hypothesis, it was recently shown that the expression levels of proteostasis network genes in *C. elegans* are responsive to nPM (12). Furthermore, the degradative pathways of the proteostasis network have also been shown to be activated in mice exposed to nPM (13). The effects of this dysregulation on the folding, or misfolding, of disease-associated proteins has never been directly tested. We therefore propose testing the hypothesis that exposure to nPM will challenge the buffering capacity of the proteostasis network, thereby reducing the efficiency of disease-associated protein folding.”
13. **Reviewer #2 pointed out that “it is not clear how the second goal and the third goal are different” and asked whether “the second goal is looking at inclusions rather than oligomers.”** That is exactly right. What is now aim “A” is examining large visible aggregates and what is now aim “B” is examining oligomers. We have also included an analysis of toxicity in with aim B because of the likely connection between oligomers and toxicity. To clarify this, we have reworded aim A as follows, “Does the exposure to nPM exacerbate the formation of large visible amyloid beta (Aβ) or polyglutamine (polyQ) protein aggregates in the genetically tractable *C. elegans?*” We have likewise reworded aim B, “Does exposure to particulate air pollution trigger an increase in polyQ or Aβ toxic oligomers in *C. elegans*?
14. **Reviewer #2 suggested that we use complete S medium instead of M9.** We had originally proposed using M9 because the one prior C. *elegans* study involving exposure to nPM utilized M9 and we wanted to be as consistent with that study as possible so as to compare results. However, it is worth noting that the prior study did short exposures, so media would have admittedly less of an effect on *C. elegans* growth and development. Therefore, we did some pilot studies in our lab to ensure that our chronic exposure in M9 does not negatively affect the animal. We found that under our proposed conditions, animals reach adulthood as expected and are phenotypically normal—meaning that they lay fertilized eggs and the eggs hatch as expected. There are no obvious developmental delays, bagging, etc. Thus, we propose sticking with the M9-based media supplemented with cholesterol and OP50.
15. **Both reviewers identified some sentences with missing or incorrect words**. Thank you for pointing these out! We have corrected them in the revised manuscript.

**Note about timeline**: Although the reviewers were comfortable with our timeline, we have revised it to reflect the fact that we are now proposing some additional experiments. We expect that the work proposed herein will be completed by the end of 2022.

I hope that the revisions to this manuscript satisfactorily address your and the reviewers’ comments and concerns. If you have any questions, or require additional information, please do not hesitate to contact me. I am excited to publish this Registered Report Protocol, as I am optimistic that it will yield some interesting and important findings.

Sincerely,

Elise Kikis

Associate Professor of Biology

University of the South
